# Small bowel metastasis from pulmonary rhabdomyosarcoma causing intussusception: a case report

**DOI:** 10.1186/s12876-019-0990-4

**Published:** 2019-05-10

**Authors:** Ke-kang Sun, Xiao-jun Shen

**Affiliations:** Clinical Medical College of Jiangsu University, Department of Gastrointestinal Surgery, Kunshan First People’s Hospital affiliated to Jiangsu University, Jiangsu, Kunshan, 215300 China

**Keywords:** Rhabdomyosarcoma, Small bowel metastasis, Intussusception, Case report

## Abstract

**Background:**

Rhabdomyosarcoma (RMS), especially primary pulmonary RMS, is an extremely rare type of soft tissue sarcoma in adults. Small bowel is an uncommon site for metastases.

**Case presentation:**

This report described an unusual case of jejunum metastasis from primary pulmonary RMS causing intussusception in a 75-year-old man. The patient consulted for 2 weeks of continuous dyspnea. Chest computed tomography (CT) demonstrated a large mass involving the left lower lobe. Transthoracic biopsy confirmed the existence of pleomorphic RMS. Immunohistochemical studies showed positive findings about desmin and MyoD1. The results of gastroscopy, colonoscopy and abdominal CT were all negative. Positron emission tomography/CT demonstrated a fluorodeoxyglucose-reactive large lesion in the left lower lobe without metastatic lesions. The patient received synchronous chemoradiotherapy. After 9 months, the patient presented with intermittent upper abdominal pain with nausea and vomiting. CT showed small bowel dilatation secondary to intussusception. The patient subsequently received laparotomy, and the intussuscepted small bowel segment was resected. Histological examination revealed pleomorphic RMS involving the mucosa, submucosa, and muscular tissues.

**Conclusions:**

RMS is highly aggressive and metastatic. The metastatic disease can rapidly progress to cause subsequent complications. The possibility of small bowel metastasis should be considered, although it is extremely rare.

## Background

Rhabdomyosarcoma (RMS) is a malignant skeletal muscle neoplasm. RMS is a common soft tissue sarcoma that occurs during childhood and adolescence, and it exists in the first 2 decades of life. By contrast, RMS is remarkably rare in adults. RMS accounts for < 1% of adult malignancies and < 3% of adult soft tissue sarcomas [1]. In addition, pleomorphic RMS is overrepresented in adult series. RMS in adults has a poor outcome compared with that in young individuals regardless of the implementation of multimodal therapies [2, 3]. RMS is commonly found in head and neck, genitourinary tract, and retroperitoneum [4]. Primary pulmonary rhabdomyosarcoma (PPR) is remarkably rare, and limited information about its clinicopathological features is reported in the literature [5–8]. RMS is highly invasive and metastatic. Metastases are mostly found in bones, bone marrows, lungs, or lymph nodes [9]. RMS metastases on the small bowel are few and far between. Herein, we present a case of jejunum metastasis due to PPR, causing intussusception in a 75-year-old man.

## Case presentation

**Fig. 1:**
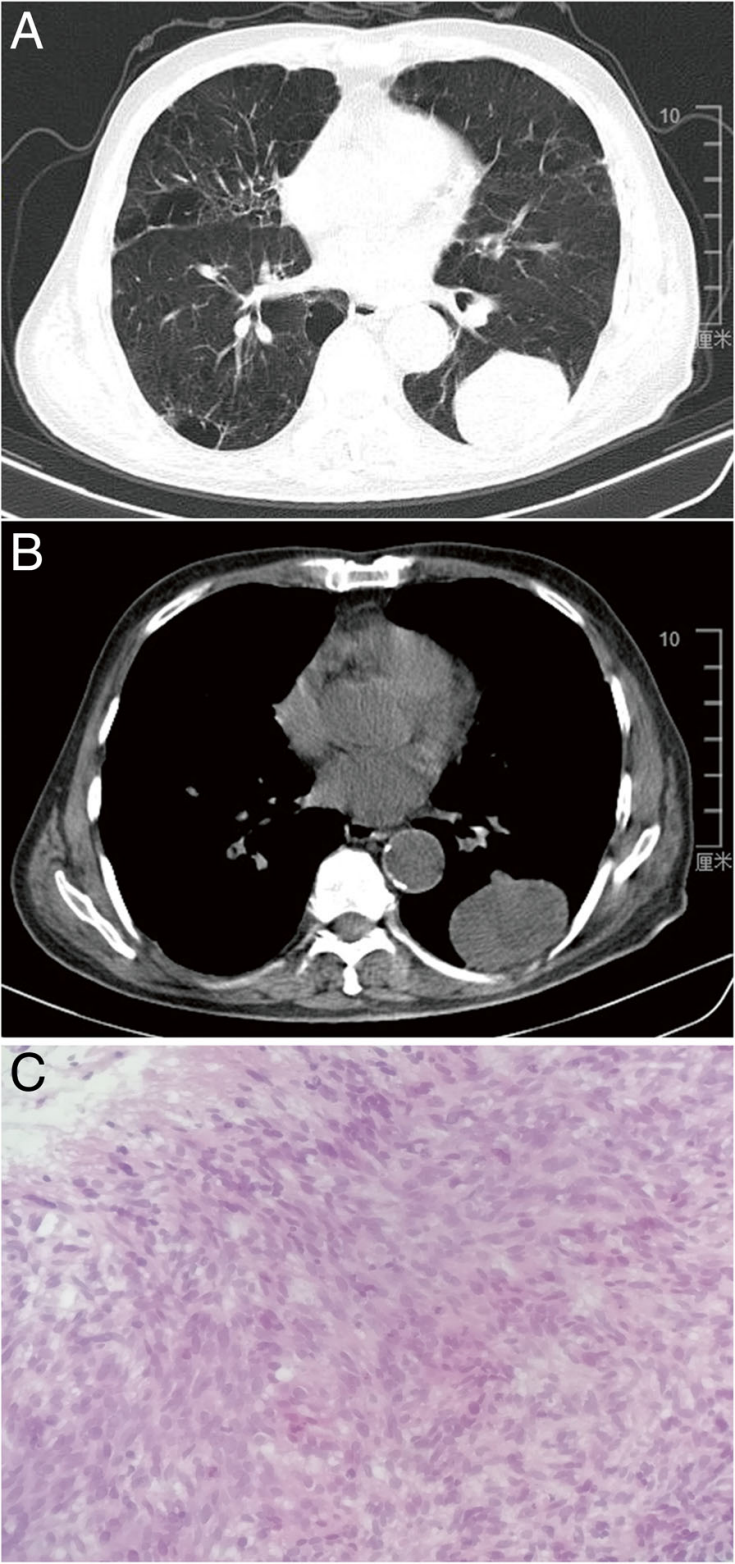
CT scan shows a large solid mass in the left lower lobe: (**a**) Pulmonary window. **b** Mediastinal window. **c** CT-guided percutaneous transthoracic biopsy showing numerous compactly clustered small malignant cells with pleomorphism (hematoxylin and eosin stain, × 100)

The subject was a 75-year-old man with chronic obstructive pulmonary disease for more than 20 years. The patient was a nonsmoker and had no history of other remarkable illnesses. The patient consulted due to 2 weeks of continuous dyspnea without fever, cough, hemoptysis, or chest pain. Lung auscultation revealed no wheezing sounds. Laboratory examinations, including complete blood count, electrolytes, renal function, liver function, and urinalysis, were negative. Chest computed tomography (CT) confirmed the presence of a large mass lesion of 5.8 cm × 4.4 cm that involves the left lower lobe (Fig. 1a and b). No lymphadenopathy or pleural effusion was observed. No mucosal lesions were identified through flexible fiberoptic bronchoscopy. CT-guided percutaneous transthoracic biopsy was conducted. Histological examination showed numerous compactly clustered small malignant cells with pleomorphism. A high mitotic rate was observed (Fig. 1c). Several rhabdomyoblasts were observed in the partial area. Immunohistochemistry showed that the cells were positive for desmin and MyoD1 and negative for c-KIT and S-100 protein, which were consistent with the diagnosis of pleomorphic RMS. Extension study was conducted through gastroscopy, colonoscopy, abdominal CT, and bone scan. The results were negative. The patient was referred for entire body fluorodeoxyglucose positron emission tomography (PET)/CT to exclude metastatic tumors. The result demonstrated a fluorodeoxyglucose-reactive large lesion in the left lower lobe with a maximum standardized uptake value of 12.8 without metastatic lesions. The patient could not bear surgical resection because of poor lung function. The patient received two cycles of vincristine/cyclophosphamide/actinomycin D chemotherapy combined with 40 Gy of intensity-modulated radiation therapy (IMRT). The patient opted to stop chemotherapy because of general weakness. After 6 months, abdominal ultrasound revealed large mass lesions in bilateral adrenal glands, which are suspected for metastasis, and the patient discontinued the therapy.

**Fig. 2:**
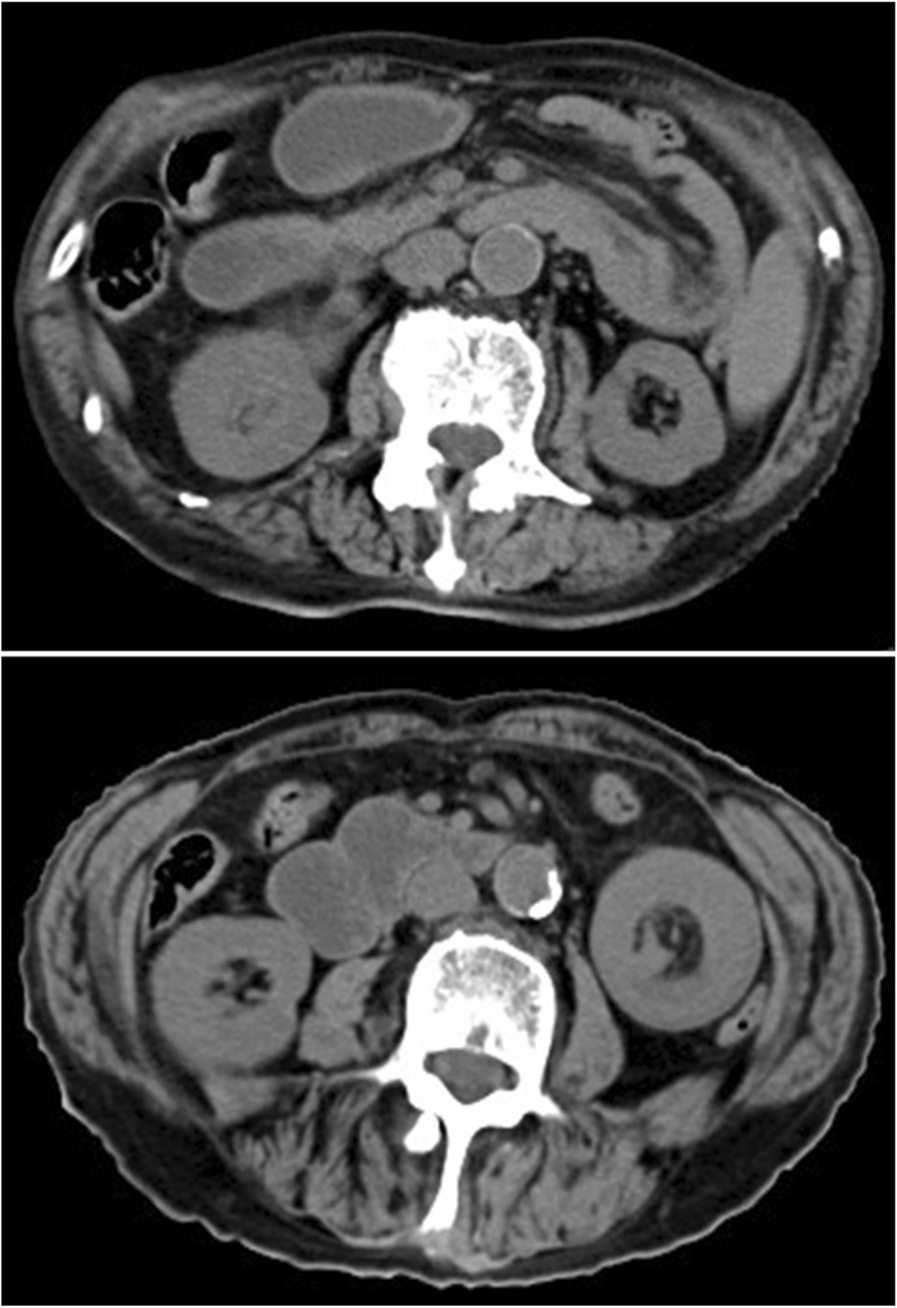
CT shows small bowel dilatation secondary to entero-enteric intussusception

**Fig. 3:**
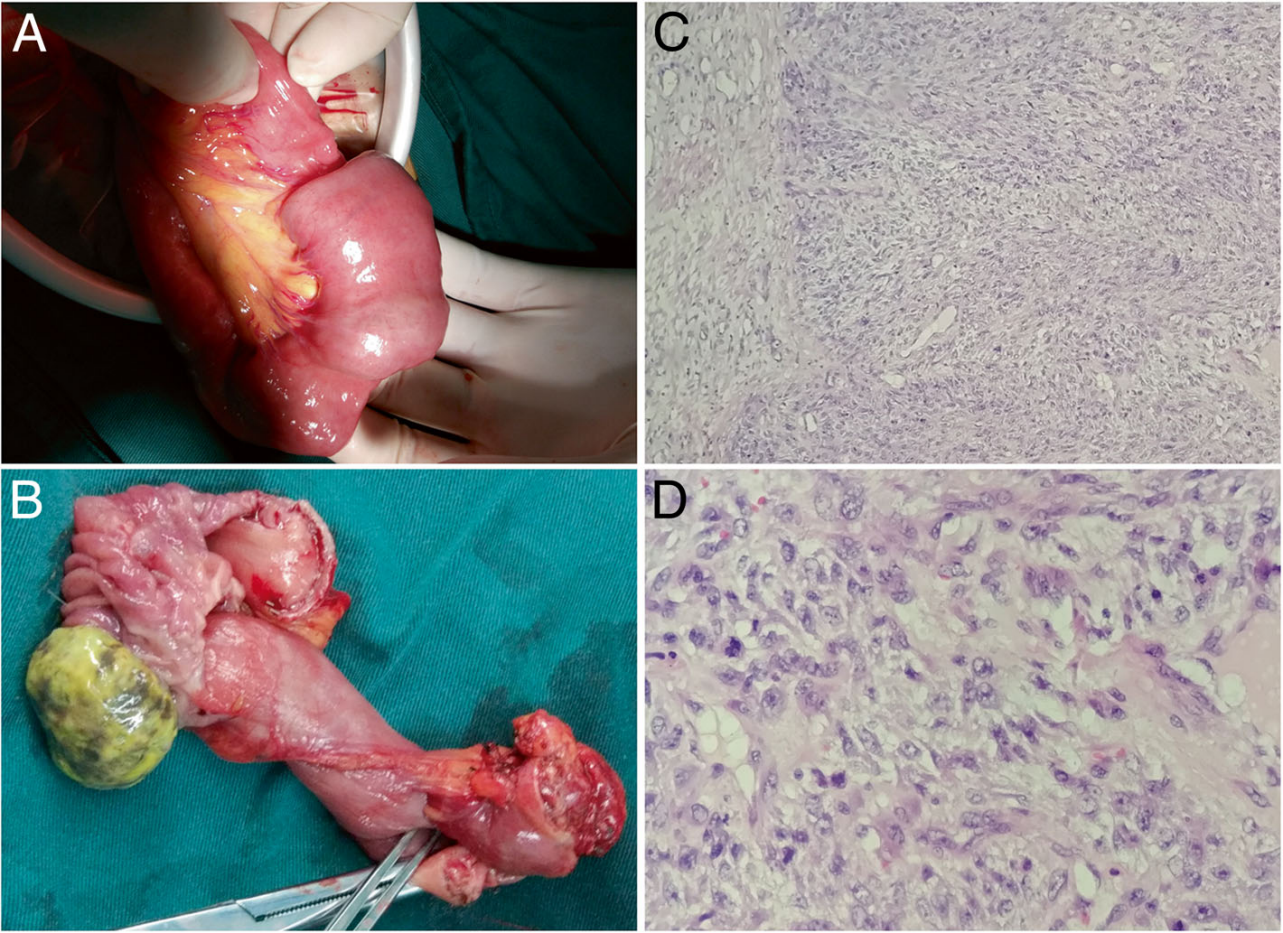
**a** Intra-operative findings of jejuno-jejunal intussusception. **b** Intraluminal mass forming the pathologic lead point. **c** Histological examination of the surgical specimen with a pleomorphism malignant cell tumor that involves the mucosal, submucosal, and muscular tissues (hematoxylin and eosin stain, × 100). **d** Several mitoses are observed in one high-power field (hematoxylin and eosin stain, × 400)

After 9 months, the patient was referred again to our hospital because of intermittent upper abdominal pain with nausea and vomiting. Physical examination showed that a large mass was palpable in the left abdomen. Bowel sounds were active. Laboratory data showed 2.1 mmol/L serum potassium. Abdominal CT showed small bowel dilatation secondary to entero-enteric intussusception (Fig. 2). The patient was given gastrointestinal decompression, potassium supplementation, and nutritional support treatment. Subsequently, laparotomy was performed, and a 15 cm segment of non-gangrenous intussusception was found in the jejunum with a 3 cm tumor forming the lead point of intussusception (Fig. 3a and b). No enlarged lymph nodes were observed in the mesentery of the affected small bowel. No other lesions were detected in the small bowel and colon. The affected segment of the small bowel was resected with end-to-end small bowel stapled anastomosis. Postoperative period was generally predictable. The histological examination of the surgical specimen revealed a pleomorphism malignant cell tumor that involved the mucosa, submucosa, and muscular tissues (Fig. 3c and d). Immunohistochemical studies were positive for desmin and MyoD1. These findings demonstrated that RMS originated in the lung. To date, the patient survived for 1 year after he was initially diagnosed and is currently under a good general condition.

## Discussion and conclusions

RMS is a common soft tissue sarcoma in the pediatric population. RMS is initially believed to originate from skeletal muscles. However, RMS is currently known to arise from early rhabdomyoblasts that can be found in various locations throughout the body with varying degrees of differentiation [10]. RMS that originates from the lung is extremely rare. Limited information is reported about the clinicopathological features of adults with RMS in the literature. To our knowledge, only a limited number of studies focus on disease processes, diagnosis or treatment in children with rhabdomyosarcomas. Older patient age at presentation, unfavorable sites of the primary tumor, the size of the primary tumor, an alveolar subtype, and the presence of regional lymph node metastasis have been recognized as prognostic factors for rhabdomyosarcoma [11]. PPR should be diagnosed only after the possibility of metastatic RMS is excluded by extensive clinical examinations. In this case, the patient could not receive contrast-enhanced CT examination because of severe allergy to iodinated contrast medium. Extension study was achieved through gastroscopy, colonoscopy, abdominal CT, bone scan, and PET/CT to exclude metastatic tumors. The results were negative.

Approximately 20% of patients have distant metastases upon presentation, which is commonly found in lungs, bones, bone marrows, lymph nodes, and rare metastatic sites [11–13]. Metastases to the small bowel are extremely rare. This condition may be due to dense lymphatic tissues in the intestines, which contain a large amount of T lymphocytes. The general route of small bowel metastasis is believed to be hematogenous in the spinal vein, retroperitoneal mediastinum, and lymphatic metastasis of the mesentery. Most small bowel metastases are caused by primary tumors, such as malignant melanoma, colon cancer, and cervical cancer [14]. The prognosis of patients with small bowel metastases is fairly poor, and most of them are found in serious complications, such as perforation, obstruction, and hemorrhage. Intussusception is rare in adults, and most cases are due to a pathologic lead point within the bowel and are malignant in more than 50% of cases, which require surgery and resection of the affected bowel segment. The common cause of malignant lesions is mainly primary tumor, and limited reports are available about intussusception secondary to small bowel metastases [15, 16].

The incidence of small bowel metastases is low, and misdiagnosis and missed diagnosis easily occur due to nonspecific symptoms. We reported the second case of small bowel intussusception caused by PPR metastasis, suggesting that the possibility of metastasis should be considered regardless of the rarity or site location [17]. Intussusception in adults should be investigated. In exploratory laparotomy, the intestinal tube should be thoroughly examined in terms of the presence of neoplastic lesions.

## Abbreviations

CT: Computed tomography
IMRT: Intensity-modulated radiation therapy
PET: Positron emission tomography
PPR: Primary pulmonary rhabdomyosarcoma
RMS: Rhabdomyosarcoma
